# Assessing the risks of SARS-CoV-2 in wildlife

**DOI:** 10.1186/s42522-021-00039-6

**Published:** 2021-04-07

**Authors:** R. J. Delahay, J. de la Fuente, G. C. Smith, K. Sharun, E. L. Snary, L. Flores Girón, J. Nziza, A. R. Fooks, S. M. Brookes, F. Z. X. Lean, A. C. Breed, C. Gortazar

**Affiliations:** 1 National Wildlife Management Centre, Animal and Plant Health Agency, Sand Hutton, York, YO41 1LZ UK; 2 SaBio, Instituto de Investigación en Recursos Cinegéticos IREC-CSIC-UCLM-JCCM, Ronda de Toledo s/n, 13005 Ciudad Real, Spain; 3 Department of Veterinary Pathobiology, Center for Veterinary Health Sciences, Oklahoma State University, Stillwater, OK 74078 USA; 4 ICAR-Indian Veterinary Research Institute, Izatnagar, Bareilly, Uttar Pradesh India; 5 Department of Epidemiological Sciences, Animal and Plant Health Agency, Weybridge, Woodham Lane, New Haw, Addlestone, Surrey KT15 3NB UK; 6 Centre de Rehabilitation des Primates de Lwiro, Kinshasa, Democratic Republic of Congo; 7 Gorilla Doctors Inc., P.O. Box 115, Musanze, Rwanda; 8 Virology Department, Animal and Plant Health Agency, Weybridge, Woodham Lane, New Haw, Addlestone, Surrey KT15 3NB UK; 9 Pathology Department, Animal and Plant Health Agency, Weybridge, Woodham Lane, New Haw, Addlestone, Surrey KT15 3NB UK; 10 School of Veterinary Science, University of Queensland, Brisbane, Queensland Australia; 11 Epidemiology and One Health Section, Department of Agriculture, Water and the Environment, Canberra, Australia

**Keywords:** SARS-CoV-2, Covid-19, Wildlife, Host-switching, Reservoirs, Risk assessment, Surveillance

## Abstract

The novel coronavirus SARS-CoV-2 likely emerged from a wildlife source with transmission to humans followed by rapid geographic spread throughout the globe and severe impacts on both human health and the global economy. Since the onset of the pandemic, there have been many instances of human-to-animal transmission involving companion, farmed and zoo animals, and limited evidence for spread into free-living wildlife. The establishment of reservoirs of infection in wild animals would create significant challenges to infection control in humans and could pose a threat to the welfare and conservation status of wildlife. We discuss the potential for exposure, onward transmission and persistence of SARS-CoV-2 in an initial selection of wild mammals (bats, canids, felids, mustelids, great apes, rodents and cervids). Dynamic risk assessment and targeted surveillance are important tools for the early detection of infection in wildlife, and here we describe a framework for collating and synthesising emerging information to inform targeted surveillance for SARS-CoV-2 in wildlife. Surveillance efforts should be integrated with information from public and veterinary health initiatives to provide insights into the potential role of wild mammals in the epidemiology of SARS-CoV-2.

## Introduction

It is estimated that there have been over 122 million cases of human infection with Covid-19 globally, with over 2.7 million deaths [1] and widespread community transmission in many countries. Early speculation on the origins of the pandemic focused on a cluster of human cases associated with a seafood market selling live wild animals in Wuhan, China [2] although evidence of other early cases in people with no contact with the market indicates the possibility of emergence from another location [3]. The causative coronavirus (SARS-CoV-2) is likely to have originated in bats (see below), although the animal species responsible for transmission to humans remains unknown. Since the onset of the pandemic, human-to-animal transmission (zooanthroponosis of SARS-CoV-2) has occurred on many occasions, in multiple countries, and involving several species, although there is to date only very limited evidence of SARS-CoV-2 infection in free-living wildlife [4]. However, it is highly likely that further cases in wildlife will emerge since many coronaviruses have a broad host range [5] with the clear possibility that spillback from humans could lead to the establishment of a reservoir of infection in wild mammals [6].

Controlling the transmission of pathogens from wild animals to humans or domestic animals is extremely challenging, and hence the emergence of a novel reservoir of SARS-CoV-2 infection in wildlife could seriously hamper effective disease control and elimination in the human population. Infection in free-living wildlife would also have substantial practical implications for management, research, rehabilitation and conservation activities [7] and could generate negative public opinion towards some species leading to persecution and disengagement from conservation initiatives. There is potential for both direct and indirect adverse effects on wildlife with implications for animal welfare, conservation and global species diversity [8]. These concerns are reflected in emerging guidance on how those who work directly with wildlife can reduce risks of SARS-CoV-2 transmission to wild mammals [7, 9]. However, there is also a need for risk reduction measures to be extended to others in the wider community who may have contact with wildlife, for example where wild animals are harvested and traded for food [10]. Such precautions are an important first line of defence, but in the face of widespread infection in the human population there is a need to also plan for the implications of SARS-CoV-2 establishing in free-living wildlife. This requires an assessment of the potential role of wildlife populations in the epidemiology of infection, and in particular, identification of those species and the circumstances most likely to lead to reservoirs of infection. Surveillance, preventative measures and contingency plans can then be developed and targeted appropriately. Based on the available evidence, the potential role of wildlife in the persistence, spread, and possible re-emergence of SARS-CoV-2 is discussed below, and a framework for dynamic risk assessment and targeted surveillance is described.

## Wildlife origins of SARS-CoV-2

SARS-CoV-2 is a betacoronavirus (β-CoV), closely related to SARS-CoV and MERS-CoV which have also caused serious outbreaks of disease in human populations. All are thought to have originated in bats [11], with evidence of intermediate or bridge hosts being responsible for transmission to humans [12–14]. Masked palm civets (*Paguma larvata*) were identified as the proximal source of SARS (Severe Acute Respiratory Syndrome) in humans [15], and dromedary camels (*Camelus dromedarius*) are a reservoir and source of MERS (Middle Eastern Respiratory Syndrome) in humans [16, 17]. Although SARS-CoV-2 may have originated in bats, its closest identified genetic ancestor (RATG13) being a β-CoV isolated from the intermediate horseshoe bat (*Rhinolophus affinis*) [13], the proximal cause of infection in humans has yet to be identified. Malayan pangolins (*Manis javanica*) have been the subject of some speculation on the basis of infection with a closely related coronavirus in animals seized in southern China [18]. Sequence analysis of the spike glycoprotein (S) of SARS-CoV-2 and related coronaviruses suggest a series of recombination events between bat and pangolin coronaviruses, may have eventually led to the emergence of this novel coronavirus [19]. However, raccoon dogs (*Nyctereutes procyonoides*), which were identified as possible intermediate hosts for the SARS pandemic of 2002–2003 [20], have also been suggested as candidate intermediate hosts for SARS-CoV-2 as experimental infection resulted in intense viral shedding [21]. Both pangolins and raccoon dogs have been found in wildlife markets in Southern China, along with many other wild mammals (wild caught and farmed) and domesticated species [22, 23]. Reports of spillover of SARS-CoV-2 from humans to companion, captive and farmed animals (see below) provide additional insights into other species that may have facilitated the jump from wildlife to humans.

## Host susceptibility

Information on the susceptibility of animal hosts to SARS-CoV-2 is emerging rapidly. There are several strands of evidence that can be used to infer the susceptibility of wild mammals, including predictions based on the characteristics of the host cell receptor to which the virus binds in order to infect cells, the demonstration of experimental infection of cell lines or of individual animals, and the confirmation of naturally acquired infection. The existence of coronaviruses with nucleotide similarity across all genes (including likely recent progenitors of SARS-CoV-2) in wild animal species may also be useful in inferring susceptibility to future infection.

Analysis of the angiotensin converting enzyme 2 (ACE2) protein, the functional receptor for the spike protein of SARS-CoV-2 in a broad range of vertebrates, has been used to predict susceptibility to infection in many mammal species [24–26]. Experimental studies using cell lines modified to express ACE2 have also demonstrated potential for SARS-CoV-2 infection in a wide variety of mammals including bats, rodents, cetaceans, carnivores and primates [27, 28] whilst ex-vivo organ culture has shown viral replication in the respiratory tissues of cattle and sheep [29]. Predictions based on such studies are subject to substantial uncertainty and neither close phylogenetic relationships amongst potential host species nor similarity in ACE2 protein sequences are sufficient for predicting susceptibility to SARS-CoV-2 [30], so this information needs to be considered alongside other evidence. Furthermore, despite in silico structural analysis and in vitro virus binding assessments being performed against ACE2 in numerous species, more information is required on the levels and locations (nasal cavity, trachea, lungs and gastro-intestinal tract) of ACE2 expression in different mammals to inform assessments of host susceptibility to SARS-CoV-2 infection.

Results from studies of experimental infection are available for several mammal species, with more information emerging daily. Such studies demonstrate susceptibility to SARS-CoV-2, albeit with varying levels of viral replication and shedding, in domestic cats (*Felis catus*) and dogs (*Canis lupus familiaris*), ferrets (*Mustela putorius furo*), American mink (*Neovison vison*), Syrian hamsters (*Mesocricetus auratus*), Roborovski’s dwarf hamster (*Phodopus roborovskii*), deer mice (*Peromyscus maniculatus*), bushy-tailed woodrats (*Neotoma cinerea*), bank voles (*Myodes glareolus*), rhesus macaques (*Macaca mulatta*), cynomolgus macaques (*M. fascicularis*), African green monkeys (*Chlorocebus* sp.), Chinese tree shrews (*Tupaia belangeri chinensis*), common marmosets (*Callithrix jacchus*), Egyptian fruit bats (*Rousettus aegyptiacus*), racoon dogs (*Nyctereutes procyonoides*), striped skunks (*Mephitis mephitis*), raccoons (*Procyon lotor*), white-tailed deer (*Odocoileus virginianus*), laboratory rabbits (*Oryctolagus cuniculus*) and mice (*Mus musculus*), and cattle (*Bos taurus*) [21, 31–47]. Experimental studies have failed to demonstrate susceptibility to SARS-CoV-2 in a range of other species including cottontail rabbits (*Sylvilagus* sp.), fox squirrels (*Sciurus niger*), Wyoming ground squirrels (*Urocitellus elegans*), black-tailed prairie dogs (*Cynomys ludovicianus*), house mice (*Mus musculus*) and big brown bats (*Eptesicus fuscus*) [34, 48]. However, experimental studies do not precisely mimic the dynamics of infection and onward transmission under natural conditions, and may overestimate susceptibility, as they often seek to maximise the likelihood of infection through the use of large volumes, and high titre inocula and/or direct instillation to target sites. The potential impact of the experimental dose on outcomes is illustrated by three separate studies on pigs (*Sus scrofa*) two of which were unable to demonstrate susceptibility [40, 43] although use of a much higher dose in the third resulted in detection of neutralising antibodies, viral RNA and live virus [49].

Naturally acquired infections of SARS-CoV-2 have been demonstrated in pet dogs, cats and ferrets in domestic settings, in tigers (*Panthera tigris*), lions (*Panthera leo*), a puma (*Puma concolor*), a snow leopard (*Panthera uncia*) and Western lowland gorillas (*Gorilla gorilla*) in zoological collections, and in farmed American mink [4, 50–56]. All such cases of natural infection have been linked to initial transmission from humans to the animals in their care. There have also been recorded cases of infection in what are described as ‘stray’ cats [53, 57] although the extent to which they were truly free-living and their levels of contact with humans are unclear. Naturally acquired infection in wild American mink was linked to a nearby mink farm where virus with an indistinguishable genotype was also isolated [58].

Direct contact with infected hosts may not be necessary for transmission of SARS-CoV-2, as studies have demonstrated the potential for coronaviruses to remain infectious for many hours on some surfaces [59] and in human faeces [60]. Recent studies have considered the possibility that contamination of aquatic systems with faeces from infected humans could provide a route for spillover into wild mammals such as raccoons and bats [61], and marine species including cetaceans and seals [62]. The detection of viral RNA in bedding, air and water samples, on the feet of a gull and from live flies collected from SARS-CoV-2 infected mink farms shows the potential for environmental contamination on these premises [63].

## Persistence and spread in wildlife

Although many viruses can jump species to infect new host populations, onward transmission and persistence are not assured, as they are affected by many factors [64]. Host susceptibility, behaviour and demography must align with pathogen characteristics to result in a successful host jumping event [65]. Hence, in order to determine the most likely species of wild mammal and circumstances whereby they might play a role in the epidemiology of SARS-CoV-2, we need to look beyond the evidence for susceptibility to infection alone.

The overwhelming majority of cases of natural infection of SARS-CoV-2 detected in non-human animals have been linked to transmission from infected humans to domestic or captive animals. While there are far fewer opportunities for transmission from humans to free-living wildlife, some activities involving direct contact may pose significant risks, such as wildlife rehabilitation, field research, practical conservation work and some wildlife-related tourism. Also, indirect transmission might occur where there are opportunities for human contamination of the environment (e.g. faeces in wastewater), supplemental food (deployed for wildlife watching, hunting or pest control purposes), urban waste or fomites (e.g. surfaces of traps used for hunting or pest control and which may be visited by animals that are either not subsequently killed or pass on infection beforehand). There will also be situations where transmission from infected humans to animals in their care creates a potential pathway for subsequent spread to wildlife (see below), hence the importance of precautionary measures related to working with domestic and farmed animals [66, 67].

Evidence to inform whether SARS-CoV-2 infection is likely to be maintained in wild animal populations is extremely scant. The animal host responsible for the initial spillover into humans remains unknown, although phylogenetic analyses suggest that the virus lineage giving rise to SARS-CoV-2 may have been circulating in horseshoe bats (*Rhinolophus* spp*.*) for at least several decades [68]. Experimental studies provide some evidence for intra-species transmission via direct contact amongst racoon dogs [21], cats [40], ferrets [43, 69], mink [31], hamsters [32], white-tailed deer [45], Egyptian fruit bats [35] and deer mice [70]. For experimentally infected cats, ferrets, mink, hamsters and white-tailed deer there is also evidence for airborne virus transmission [31, 32, 40, 45, 69].

Natural transmission of SARS-CoV-2 has taken place amongst farmed mink following initial introduction from farm workers [52, 53]. Transmission amongst separately housed mink suggests spread by fomites, respiratory droplets or aerosols [53] which is consistent with widespread detection of viral RNA during environmental sampling of infected premises [63]. Mink farms provide the only current source of evidence for maintenance of naturally acquired infection in an animal population and spillback to humans [71]. The housing of mink at unnaturally high densities and the spatial structure of farms may facilitate rapid spread and persistence of the virus in these captive populations [71]. In contrast, animal to animal transmission within groups of captive felids and primates could not be confirmed following outbreaks of SARS-CoV-2 in zoos, but seems plausible given evidence for virus shedding in faeces and respiratory secretions [55, 56].

The scale and widespread distribution of infection in the human population means that the current role of wildlife in the global epidemiology of SARS-CoV-2 is likely to be negligible. However, this will likely change over time, with the significance of a reservoir of infection in wild mammals potentially increasing as community transmission in human populations is reduced in the face of effective control measures. In such situations, the implications of spillback from a reservoir of infection in wildlife populations would be more significant. It is also possible that the circulation of SARS-CoV-2 in a wild animal population might lead to the evolution of phenotypic variants as the virus adapts to new species, with implications for onward transmission and control in human populations [6].

## Potential wildlife reservoirs

While it is not possible to assess the risks of SARS-CoV-2 in all wild mammal species, a priority list of species groups for initial consideration can be assembled using available evidence. Below we discuss the potential for exposure, onward transmission and persistence of SARS-CoV-2 in an initial selection of wild mammals, which could on the basis of current evidence, be considered of particular relevance.

### Bats

Many of the known coronaviruses appear to have a bat origin [72]. The closest known ancestor of SARS-CoV-2 (RATG13) was isolated from a rhinolophid bat in southern China [11] and other closely related coronaviruses have been found in this family elsewhere in south-east Asia [73]. The wide diversity of coronaviruses found in bats, suggests high potential for viral evolution in these species, and indicates the potential for recombination of SARS-CoV-2 with other coronaviruses [74].

Although recorded instances of transmission of viruses from humans to bats are rare and onward transmission has not been recorded [75], systematic surveillance has been lacking and so instances may have gone undetected. Opportunities for pathogen transmission between bats and people may occur via the actions of bat carers, veterinarians, field ecologists, conservation and research workers, or through inadvertent contact between bats and humans arising from deforestation, mining, ecotourism and food production [76]. Guidance has been published for reducing the risk of SARS-CoV-2 transmission from humans-to-bats in field research [77] and in bat rescue and rehabilitation centres [78]. Humans may come into contact with bat faeces where bats roost or hibernate in buildings.

The high population density at which many bat species roost and high population sizes are likely to facilitate pathogen transmission and persistence, although the existence of related viruses in many bat populations may confer a level of immunity to SARS-CoV-2 and so reduce the likelihood of persistence. Coronaviruses in bats typically have a narrow host range but there is genetic evidence of many host switching events, and that this process contributes to coronavirus evolution [79, 80].

Should SARS-CoV-2 enter a previously uninfected bat population due to transmission from humans the impacts would be highly uncertain [75], and given their nocturnal and often cryptic behaviour, population-level effects would be unlikely to be detected in many situations. However, detecting infection in a bat population could precipitate an erosion of the perceived biodiversity value of bats, resulting in loss of current protections and deliberate persecution. Even the perception (with no evidence) that they are involved in the transmission of SARS-CoV-2 to humans has resulted in the killing of bats in several countries according to anecdotal reports [81]. These events are not only concerning for bat conservation given that so many species are of threatened or unknown status [82] but may also increase the risk of spillover of other pathogens to humans.

Surveillance for SARS-CoV-2 in bats could be targeted at animals coming into very close proximity with humans, such as those undergoing rehabilitation prior to release, those maintained in captivity or captured for research purposes. Priority bat populations for surveillance could include those that roost in buildings or public spaces (e.g. urban parks) where inadvertent contact with humans is more likely to occur. There is some evidence that SARS related coronaviruses are more strongly associated with bat species in the Old-World suborder Yinpterochiroptera and those in the genus Rhinolopus in particular [75] suggesting that these groups could be prioritised, although available surveillance and sampling data is geographically and taxonomically biased by specific research activities.

### Felids

Observations from experimental and natural SARS-CoV-2 infections in animals clearly suggest relatively high susceptibility amongst felids (Felidae family) with human-to-feline transmission recorded in pet cats [54, 58, 83] and captive wild species such as tigers and lions [55, 84], and evidence for transmission amongst domestic cats via direct contact [85] and from airborne virus [40]. SARS-CoV-2 antibodies have been detected in ‘stray’ cats in Wuhan during the Covid-19 outbreak consistent with human-to-cat transmission occurring outside the domestic setting [57], although possible cross-reactions with other coronaviruses need to be fully assessed. Also, the observation of infected ‘stray’ and pet cats in the vicinity of infected mink farms has raised the (as yet unconfirmed) possibility of inter-species transmission [53, 63]. In addition, cats may theoretically be exposed to infection through interactions with their prey such as rabbits, rodents and bats.

Domestic cats are the most abundant felids, reaching densities in excess of 2000 animals km^2^ in urban areas [86], and their proximity to humans, mobility and social interactions provide ample opportunities for inter-species pathogen transmission. Although ‘stray’ and truly feral domestic cats typically have less contact with humans, they may nevertheless be exposed to human-derived infection via fomites in residential areas and farm environments for example. Social interactions amongst colony-living feral cats may be conducive to intra-specific transmission, although there is no current evidence for SARS-CoV-2 maintenance within cat populations, nor for transmission from infected cats to humans. Nevertheless, on the basis of available evidence, surveillance for SARS-CoV-2 in felids could target free-living domestic cat populations, particularly where they are abundant in urban environments or in the vicinity of other potential sources of infection such as mink farms. In contrast, wild felid species tend to be solitary, are far less abundant and seldom come into contact with humans and urban environments, so would not be expected to contribute to virus persistence. However, rare and endangered species could be at risk of exposure from infected humans through research and conservation programmes. Domestic cats naturally infected with SARS-CoV-2 have often been reported as showing none or only mild clinical signs, although some instances of more serious disease have been reported [54, 83, 85]. Whereas only mild respiratory signs accompanied infection in captive tigers and lions [55, 84]. It is therefore unclear whether infection would adversely impact wild felid populations.

### Canids

There are several instances of SARS-CoV-2 infection in domestic dogs associated with presumed transmission from humans [50, 87]. As clinical signs in domestic dogs appear generally mild with only limited viral shedding [40, 87], the evidence to date suggests that onward transmission amongst dogs or to other species is unlikely. However, this may not necessarily hold true for wild canids. For example, raccoon dogs have been shown to be susceptible to experimental infection and capable of onward transmission of SARS-CoV-2 [21]. The susceptibility of other wild canids such as foxes, and jackals is unknown, although analysis of the ACE2 receptor predicts that red foxes (*Vulpes vulpes*) would be susceptible [88]. Onward transmission amongst free-living canids would be most likely where they are group-living (e.g. family groups and packs) or reach relatively high densities such as in urban feral dog populations.

Perhaps the greatest opportunities for close interactions between humans and free-living canids involve feral and community-owned dogs, whereas human contact with wild species is likely to be mostly restricted to hunting or pest management. However, many wild canid species are opportunistic scavengers (e.g. jackals, red foxes) which may bring them into contact with potential sources of infection such as mink farms. In China raccoon dogs are farmed for their fur and hence similar to mink farms in potential for spillover of SARS-CoV-2 from infected workers to captive animals, followed by onward spread and spillback to humans [21].

### Mustelids

There have been many cases of SARS-CoV-2 in farmed mink with infections reported from Europe and North America [71]. Transmission from humans to mink, onward spread amongst mink and spillback to humans have been confirmed [52, 53], including the emergence of a new variant of SARS-CoV-2 in communities adjacent to mink farms in Denmark [71]. The first confirmed case of infection in a free-living wild animal was an American mink captured in the vicinity of an infected mink farm in Utah, USA [58] where several infected escaped mink were also found [89]. Infection has since also been reported in two free-living American mink captured during feral mink eradication activities in Spain [90]. Such situations may arise because of escaped animals establishing in the locality or when wild animals attracted by sources of food, and in the case of wild mink by the scent of con-specifics, come into close proximity with captive animals, fomites and airborne virus from contaminated bedding and dust [71]. Surveillance of wild mustelids (and other carnivores) in the vicinity of mink farms is therefore an effective means of targeting wildlife at relatively high risk of exposure to SARS-CoV-2 [71]. Ferrets are also farmed (for the pet trade and medical research) which may provide opportunities for virus introduction from infected people, and as experimental studies have shown, onward spread may occur amongst in-contact animals [43]. Ferrets are kept as pets and in some parts of the world (e.g. UK, Spain) are used for hunting rabbits which combines close contact with their human keepers with the potential for escape into the wild and interaction with wild mammals. SARS-CoV-2 infection has been confirmed in ferrets kept for hunting purposes in Spain, although persistence was deemed unlikely in the small groups involved [51].

It has been suggested that wild mink could be a reservoir species [52], although population density and the frequency of contact in the wild are far lower than in captivity. Studies of SARS-Cov-2 outbreaks in farmed mink have revealed evidence of rapid virus evolution, together with onward transmission to humans [70]. Rapid mutation of the virus and the potential emergence of host-adapted variants are probably less likely in wild populations, but cannot be ruled out as indicated by the identification of amino acid polymorphisms that might influence function of the spike protein in low numbers of experimentally infected ferrets [91].

Scavenging and predation on other potentially susceptible mammals could provide opportunities for spillover into wild mustelid populations, although in most species their social organisation is likely to mitigate onward transmission. Wild mustelids generally occur at relatively low densities, and with few exceptions are largely solitary, with contact amongst adults being typically confined to the breeding season, thus limiting opportunities for onward transmission and persistence. One notable exception is the European badger (*Meles meles*) which lives in social groups of varying size across its wide geographic range and reaches high densities in some locations (up to 38/km^2^ recorded in southern England [92]). In the UK and Ireland they may be the subject of management interventions (e.g. trapping for culling, vaccination or relocation of their den sites), all of which might facilitate virus transmission and could provide opportunities for surveillance.

### Great apes

Captive and wild great apes (Hominidae) are highly susceptible to many viral pathogens of humans [93, 94] with respiratory infections of human origin causing disease that poses a significant and growing threat to the conservation of wild populations across Sub-Saharan Africa [95]. The characteristics of ACE2 predict high susceptibility to SARS-CoV-2 amongst primates, particularly Old-World species [24, 96] and this is borne out by the results of experimental studies [36, 39, 41] and naturally acquired infection in captive gorillas [56].

Risks of exposure of wild primates to SARS-CoV-2 could arise wherever they have direct or indirect contact with humans from local communities, which is not uncommon as they can become highly habituated to human activity. Interactions with humans occur where deforestation increases human access and displaces primate populations, wherever they are captured and traded, and as a result of rehabilitation, research, conservation and tourism. Also, most important primate conservation areas are surrounded by densely populated human settlements where interactions with people may be inevitable, and where local community transmission of SARS-CoV-2 is not well documented.

Hence, there is ample evidence for the vulnerability of endangered great ape populations to SARS-CoV-2 transmission from humans, and onward spread would be facilitated by their highly social behavior, but the potential impacts on their health are less predictable. To date SARS-CoV-2 infection in great apes has only been confirmed in one group of captive Western lowland gorillas [56], all of which developed clinical signs, mostly mild and consisting of coughing and nasal discharge, which resolved after a few days. A 48 year old male with an underlying heart condition developed a pneumonia but recovered after veterinary treatment. Although the virus is assumed to have been introduced by a zoo keeper, subsequent  transmission amongst the gorillas cannot be ruled out as virus was isolated from faeces and detected in a nasal swab [97]. The health impacts of SARS-CoV-2 could be more serious in wild gorillas as they are subject to co-infections and physiological stressors that are absent in captive animals under veterinary care. It is also difficult to predict the potential impacts of SARS-CoV-2 infection in other wild primates, so given past experience of human-derived respiratory infections and the precarious conservation status of wild great ape populations, the potential for adverse health and population impacts should be considered high and risks managed accordingly. This might potentially involve strict health surveillance and vaccination of tourists, researchers and conservation workers who may come into close contact with primates, improved hygiene and sanitation, use of protective equipment and safe distancing, with quarantine measures where management interventions require moving animals [98]. A vaccine developed for use in great apes has been administered to captive bonobos (*Pan paniscus*) and orangutans (*Pongo* sp.) [97] and this approach could also be applied during rehabilitation and in habituated free-living primates.

### Rodents

Host cell receptors in several rodent species exhibit high binding affinity for the SARS-CoV-2 spike protein, particularly those in the Cricetidae family which includes hamsters, voles and New World rats and mice [24, 26]. Experimental studies have demonstrated susceptibility in Cricetidae species, with evidence of onward transmission to in-contacts for deer mice and hamsters [32, 34]. In contrast the house mouse (an Old World rodent) has been predicted to have low susceptibility to SARS-CoV-2 on the basis of the binding affinity of ACE2 [24], and this is consistent with failure to establish experimental infection [34]. However, a study of laboratory mice showed that although they were not susceptible to experimental infection with an ancestral strain of SARS-CoV-2, two variants that have recently emerged in humans (so called ‘variants of concern’ VOC) resulted in virus replication in the lungs [46]. This is an important finding as it demonstrates the potential for the host range of SARS-CoV-2 to expand as new variants emerge.

Rodents are the most abundant and diverse group of mammals, and are known hosts of a range of alpha and beta-coronaviruses [99, 100]. Many species (particularly rats and mice) live in very close proximity to humans, their companion animals and livestock, often sharing living spaces, providing many opportunities for transmission. Surveillance for SARS-CoV-2 in rodents could prioritise species with these habits, that are of known or suspected susceptibility, and exhibit life-history traits that are conducive to the maintenance of infection (e.g. high fecundity and early sexual maturation [101]).

### Cervids

Cervids have been identified as potentially susceptible to SARS-CoV-2 infection on the basis of the binding affinity of their ACE2 receptors [24]. However, susceptibility to SARS-CoV-2 infection has to date only been demonstrated in white-tailed deer which developed subclinical infection with onward transmission to in-contact animals following experimental challenge [45].

Transmission of SARS-CoV-2 from humans to cervids could potentially occur at deer farms and zoos. As farmed deer may interact with wild deer and other species or escape where biosecurity measures are inadequate, this might provide opportunities for the spread of infection to free-living wildlife. The extensive herding of reindeer (*Rangifer tarandus*) in parts of northern Europe provide many potential interfaces for transmission of infection from humans. Wild deer could also potentially become exposed to infection from humans through contact with contaminated feed put out by hunters. Many cervid species live in groups (of related or same sex individuals) during at least part of the year but can congregate in larger numbers in response to food availability. These patterns of sociality provide potential opportunities for onward spread and persistence of the virus, whilst interactions with livestock (e.g. on grazing land or at waterholes) or predation could facilitate inter-species transmission.

## Dynamic risk assessment and surveillance

The sections above describe some of the potential pathways for human to wildlife transmission and onward spread of SARS-CoV-2 in potentially susceptible wild mammals. Although many evidence gaps currently limit our ability to accurately assess these risks, initial assessments can provide a useful framework on which to build as new data emerges. Integration with surveillance activities will permit these approaches to inform one another in a dynamic iterative process, whereby the outcome of risk assessments helps direct surveillance which in turn provides crucial data to underpin the assessment of risks.

**Fig. 1:**
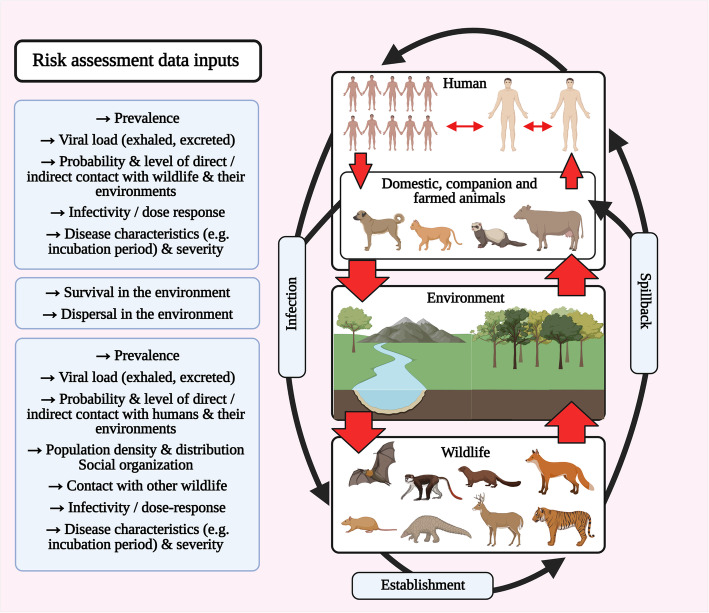
Potential pathways of SARS-CoV-2 transmission in a multi-host system and sources of data for the assessment of associated risks

There is an urgent need to develop frameworks to assess the risk of SARS-CoV-2 becoming established in wild mammal populations and onward transmission to humans [6, 102]. From a One Health perspective, the dynamics that need to be considered in such assessments relate to infection in the human population, in domestic animals and wildlife, and the associated probabilities of transmission to wildlife from a human or domestic animal source, the likelihood of persistence in the wildlife population and potential for transmission back to humans or domestic animals (Fig. 1). Also, the virus could potentially be transmitted amongst different populations of domestic or wild animal hosts, and all transmission pathways may potentially be direct or indirect (via the environment).

Owing to the many data gaps and deficiencies in our understanding of the epidemiology of SARS-CoV-2, it is essential that uncertainty and variability are captured within any risk assessment framework and communicated. This could include uncertainty relating to not only the quality of the data used to assess the risk (e.g. published literature vs expert opinion, the biological relevance of experimental studies), but also model uncertainty as many of the pathways of SARS-CoV-2 transmission between humans and wildlife may be speculative. Of the limited number of studies undertaken to date, two qualitative assessments of the risks of SARS-CoV-2 becoming established in bat populations concluded that the overall risks were ‘low’, but that uncertainty was high in part because of the absence of data on the frequency and context of human-bat interactions, susceptibility to infection and the capacity for subsequent viral shedding in bats [103, 104]. These are likely to be typical outcomes of assessments for many species, but such exercises are nonetheless useful as they identify important areas of data shortfall which if addressed will reduce levels of uncertainty and help to inform surveillance efforts.

Pathogen surveillance in wildlife populations is challenging owing to the difficulties in undertaking representative and unbiased sampling, the practicalities of obtaining samples from free-living animals, determining the most cost-effective sampling design, the limitations of diagnostic test performance and lack of validated tests in wildlife species. The choice of surveillance approach should be strongly influenced by the primary purpose, which may be early detection of infection, demonstration of absence of infection, determination of presence or mapping the distribution of infection. The activities undertaken to suit a particular purpose will depend on various factors including the expected prevalence of infection and severity of disease (e.g. can mortality or morbidity be useful indicators of infection), the distribution, abundance and accessibility (e.g. how cryptic they are) of the target species, the availability of financial resources, trained personnel and facilities for sample collection, transport, storage and testing. Programs for general surveillance to investigate mortality events in wildlife are not present in many countries and where they are, they will have limited sensitivity particularly for the detection of early stage SARS-CoV-2 infection and the early incursion of infection into a population when prevalence may be low.

Indiscriminate surveillance for SARS-CoV-2 in wildlife is unlikely to be an effective approach, instead surveillance should be targeted to generate outputs that can inform the evidence base and management options [105, 106]. Surveillance in wildlife should also be integrated with wider public and animal health strategies so it can inform our understanding of transmission pathways amongst these different populations [105, 106]. It is also essential that all necessary precautions are taken to ensure that surveillance activities themselves do not expose wildlife to risks of infection, and that public messaging related to SARS-CoV-2 in wildlife is managed so as to avoid wider welfare impacts [107].

**Fig. 2:**
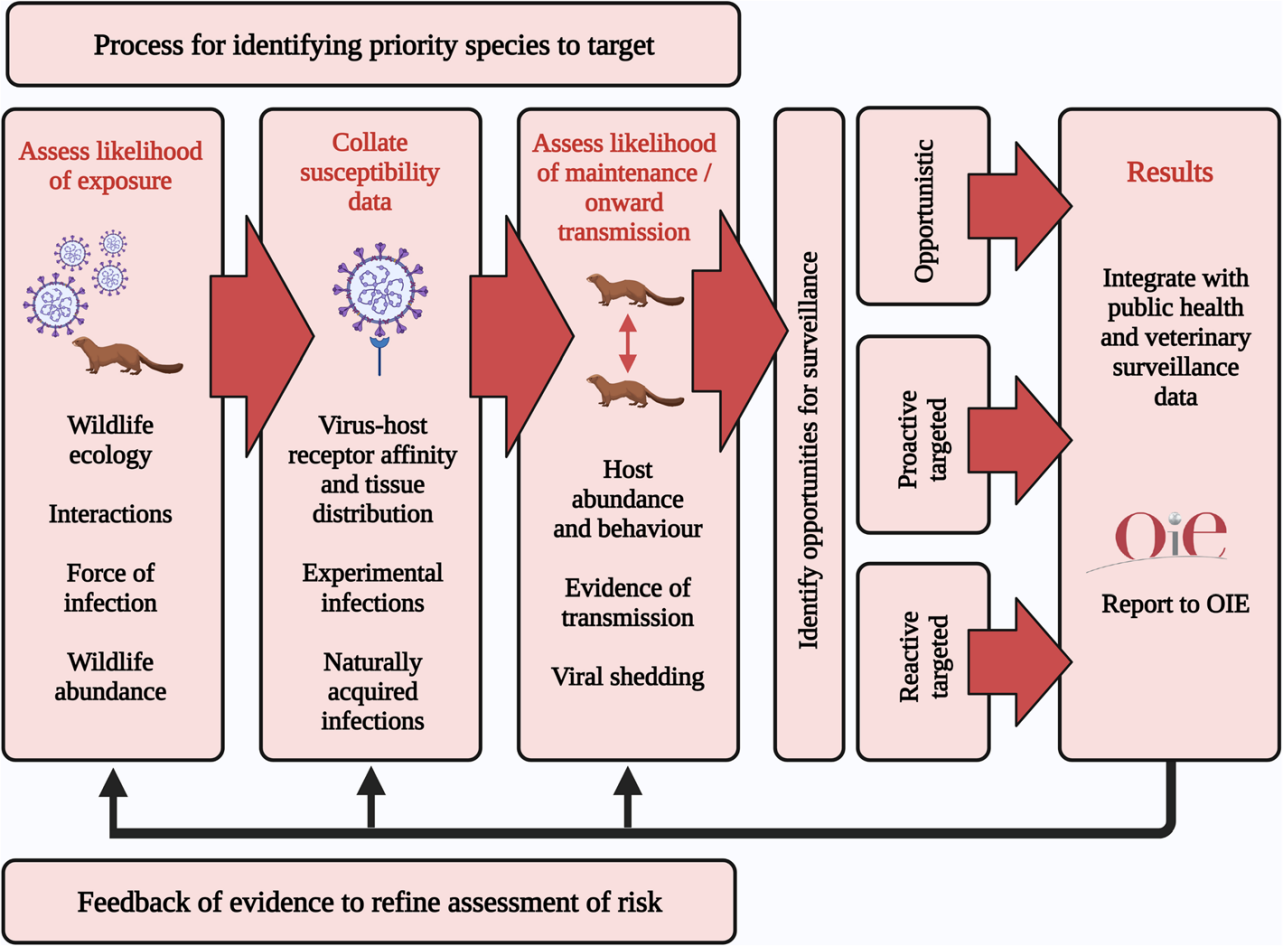
Conceptual framework for the integration of information to inform surveillance for spill over of SARS-CoV-2 infection from humans to wildlife

A broad framework for SARS-CoV-2 surveillance in wild mammals will involve the integration of information on the likelihood of exposure to infection from humans, host susceptibility and the potential for onward spread and persistence, in order to identify candidate species, populations and opportunities for targeted sampling (Fig. 2). The potential for exposure of wildlife to an infection that is widespread in the human population and has already spilled over into companion and captive animals must be considered significant, but will vary widely amongst species, being greatest for those that are sympatric or have particular interactions with humans and susceptible companion and captive animals. These will include species that share urban environments with humans and their companion animals, are the subject of activities involving close interactions with people (e.g. veterinary intervention, conservation, research, hunting, pest control etc.) and are associated with high risk environments (e.g. in the proximity of mink farms or urban habitat). Such circumstances may also provide opportunities for wildlife to human transmission.

The likelihood that exposure will lead to infection in wild species can be informed by the existing evidence for susceptibility (derived from the sources described above). Susceptible species that are at risk of exposure can therefore be considered as potential priorities for surveillance. Evidence that suggests the capacity for persistence of infection in such species may further refine this prioritization process. The abundance and social behavior of wild hosts and their capacity for viral shedding will be critical determinants of the potential for spillover to lead to onward spread and persistence. However, it is important to recognize that simple relationships between density and infection dynamics may be confounded by host behavior and indirect transmission routes [108]. Similarly, aggregations such as colonies of roosting or hibernating bats and the social groups of some carnivores and primates might be expected to enhance transmission, although social structure in wild mammal populations can also limit epidemic spread [109]. Hence, the ecological characteristics of potential host populations will need to be carefully considered during the assessment of risk. For some species with high risks of exposure and maintenance of infection, there will be little or no information on their potential susceptibility, in which case the need for further research in this area is indicated.

Once priority species for surveillance have been identified then opportunities for sampling can be determined. These may fall into three broad categories; opportunistic, proactive targeted sampling or reactive targeted sampling. Opportunistic sampling can take place wherever species of interest are being captured and/or handled for other purposes such as wildlife rehabilitation, veterinary care, management and field research. These activities may themselves represent potentially high risks of exposure, so the approach is not entirely untargeted. This is likely to be a cost-effective strategy and can be stratified to prioritise surveillance in locations of particular interest (e.g. urban areas). Another approach is to carry out proactive targeted surveillance in locations where the risks of exposure of wildlife to SARS-CoV-2 are potentially high, such as around mink farms [71]. In contrast, reactive targeted surveillance would be triggered by events that signal a local increase in the risks of exposure to wildlife, such as an outbreak of infection at a mink farm or detection in a wild animal via opportunistic sampling. The species groups listed above, particularly those for which onward transmission has been confirmed, could be considered as candidates for opportunistic sampling in the first instance, and for risk assessments to determine the case for more targeted surveillance. Given our limited knowledge of susceptibility to SARS-CoV-2 in many wild species, proactive and reactive sampling of wildlife in high risk situations could be broadened beyond just the prioritised species. A surveillance programme that is able to combine and switch between all three approaches is likely to have the flexibility required to respond to epidemiological changes and the emergence of new evidence. Any surveillance strategy for SARS-CoV-2 in wildlife should include processes for the notification of confirmed cases to the OIE through the World Animal Health Information System (WAHIS) [110] and for the integration of results (at a local, regional or national scale) with epidemiological data collected by public health and veterinary services [105].

Surveillance for SARS-CoV-2 in wildlife could involve testing serum for specific antibodies against SARS-CoV-2, detection of viral RNA or proteins in clinical samples or tissues, or testing for the presence of viral RNA in the environment. Serum antibodies against SARS-CoV-2 can be detected by ELISA and by serum neutralization. ELISAs are convenient for large numbers of individual animals but require validation, including determination of diagnostic sensitivity and specificity, for the species in which the test is to be applied. Indirect ELISAs require suitable secondary antibodies for each species, but these are not always readily available for wild animals. Moreover, cross-reactions with other coronavirus infections are likely to limit the specificity of all ELISAs. Alternatively, serological techniques that do not require a secondary antibody, such as competitive ELISAs, two-step incubation ‘sandwich’ ELISAs, or serum neutralization tests can be used to overcome this limitation [71]. Serology has the limitation of detecting only historical infection with limited temporal accuracy, but this can make it the method of choice for screening at the population level.

Testing individual animals for SARS-CoV-2 infection by PCR (polymerase chain reaction) for virus nucleic acids provides confirmation of current infection status. Suitable samples to collect from live animals are oral (preferred) and rectal swabs. Upper respiratory tract (e.g. nasal turbinates and trachea) and lung tissue are appropriate samples to collect from carcasses [71, 106]. If the viral load is sufficiently high then it may be possible to culture live virus from samples. Genetic sequences will provide valuable phylogenetic information on the relatedness of viral lineages and may allow the inference of transmission pathways. Rapid antigen tests can detect specific viral proteins through colorimetric reactions, and in principle can also be used in wildlife, although this will require further research.

Subjecting environmental samples to PCR testing for SARS-CoV-2 RNA has the potential to detect recent infection in large groups of individuals and has proven useful in surveillance of human populations via the testing of surfaces [111], sewage and wastewater [112]. Similarly, approaches such as testing faeces under bat roosts [113] or sampling and testing water or surfaces could be considered for wild animal populations [71].

The inter-species infection dynamics of SARS-CoV-2 are highly complex compared to previous zoonotic CoVs due to its rapid global spread through the human population, high prevalence in some communities and broad host range which present widespread opportunities for cross species jumping. Hence understanding the epidemiology of this virus and managing its spread requires a truly One Health approach. In this regard, molecular epidemiological surveillance will be particularly valuable to identify transmission pathways across this host range and the genetic changes and novel adaptations linked to cross-species transmission [19]. This is of particular importance given the recent observation that SARS-CoV-2 Variants of Concern (VOC) such as B.1.1.7 can spill over into animals [46].

## Conclusions

Although the SARS-CoV-2 pandemic is currently being driven by human-to-human transmission with no evidence that domestic or wild animals are playing an important role, this may not remain the case. The establishment of a reservoir of infection in a wild animal population would pose a significant risk to public health if it had potential to spillback into communities where the burden of infection had been reduced through control measures and/or herd immunity. Furthermore, sustained transmission in a wild host population would provide an opportunity for evolutionary adaptation of the virus, which could potentially (positively or negatively) influence transmission dynamics and the effectiveness of diagnostics and vaccines. Risk assessment and targeted surveillance are important tools for improving our understanding of the potential role of wildlife in the epidemiology of SARS-CoV-2, but developing systematic approaches is challenging given the paucity of available evidence. Here we have described a broad framework for collating the available epidemiological and ecological data to inform the process of prioritising species for surveillance and three modes of sampling wildlife that provide cost-effective and targeted options.

The SARS-CoV-2 pandemic serves as a powerful demonstration of the links between the health of wildlife, domestic/farmed animals and humans. Hence, we stress the need to take a One Health approach to investigating the potential role of wild mammals in the continuing epidemiology of SARS-CoV-2. In particular, this should involve the integration of targeted surveillance and the dynamic assessment of risks to animal and human health. Importantly, this need extends beyond the current pandemic, and speaks to the wider requirement for a proactive approach to assessing the dangers of diseases emerging from wildlife [10]. Hence we reiterate the call by Olival et al. [75] for the development of an adaptive framework for surveillance and risk assessment of other coronaviruses in wildlife, domestic animals and human populations at high risk of exposure, so that in the future we may be better prepared to prevent and control their potential impacts on human and animal health.

## Data Availability

Not applicable.
